# How Women Drink: Epidemiology of Women’s Drinking and Problem Drinking

**Published:** 1994

**Authors:** Sharon C. Wilsnack, Richard W. Wilsnack, Susanne Hiller-Sturmhöfel

**Affiliations:** Sharon C. Wilsnack, Ph.D., is Chester Fritz Distinguished Professor and Richard W. Wilsnack, Ph.D., is a professor at the Department of Neuroscience, University of North Dakota School of Medicine, Grand Forks, North Dakota. Susanne Hiller-Sturmhöfel, Ph.D., is associate editor of Alcohol Health & Research World

## Abstract

More than 50 percent of women in the United States drink alcohol. In general, they abstain more and drink less frequently than men. Women’s drinking behavior is determined by demographic characteristics, such as age, ethnicity, employment, and marital status, as well as by individual and environmental risk factors.

Gender is a strong predictor of drinking behavior. According to a 1990 national survey of drinking by American adults (Midanik and Clark 1992), men are more likely than women to drink at all (71 percent of men and 59 percent of women drink any alcohol) and are more likely than women to be heavy drinkers (21 percent of men and 6 percent of women have two or more drinks^1^ daily).

Data from 39 longitudinal surveys^2^ in 15 countries have confirmed these gender differences. In every country and every age group, men drink larger quantities, drink more frequently, and report more drinking-related problems^3^ than women (Fillmore et al. 1991). However, women’s drinking behavior can vary significantly among countries and within a country over time.

The gender differences in drinking and heavy drinking rates are paralleled by gender differences in the prevalence of the clinical diagnoses of alcohol abuse and alcohol dependence. (The terms “alcohol abuse” and “alcohol dependence” are used as defined in the *Diagnostic and Statistical Manual of Mental Disorders, Fourth Edition* [American Psychiatric Association 1994].) In all studies conducted, more men than women were diagnosed with these disorders, but the male-to-female prevalence ratios vary considerably among studies. For instance, Kessler and colleagues (1994) found three times more alcohol-abusing and alcohol-dependent men than women, whereas Robins and colleagues (1988) reported male:female ratios ranging from 4:1 to 8:1. These differences in ratios are due in part to interview questions in some surveys that emphasize drinking behavior or drinking problems more common in men; however, why the male-to-female prevalence ratios are *so* variable across studies remains to be determined.

The lower rates of alcohol use and abuse among women than among men do not mean that women’s drinking behavior is socially inconsequential and need not be studied in detail. On the contrary, research on women’s drinking is important for at least three reasons. First, until the 1970’s, alcohol research of all forms—epidemiological, clinical, and experimental—either excluded women entirely or focused disproportionally on men. And although research on women and alcohol has increased substantially since then, significant gaps in knowledge still exist (see Gomberg and Nirenberg 1993; Wilsnack and Beckman 1984).

Second, the biomedical and other consequences of women’s alcohol use may be greater than their lower consumption levels seem to imply. For example, as a rule, a woman’s susceptibility to the physiological consequences of alcohol abuse is higher than a man’s. Women reach higher blood alcohol concentrations than men from the same weight-adjusted levels of consumption and may develop liver disorders after lower levels of regular alcohol consumption and earlier in their drinking careers than men (e.g., Hill in press; for more information, see sidebar by Deal and Gavaler, pp. 189–191).

Third, understanding more about women’s drinking can increase knowledge about the factors that influence drinking behavior and drinking-related problems more generally. Trying to understand why women as a group drink less than men, but also why some women drink more than other women, can help to clarify how gender and gender roles (i.e., social expectations about how women and men should behave) affect the drinking behavior of both women and men.

In this article, we review general trends in women’s alcohol consumption over the last 20 years and present data from a 10-year longitudinal study conducted between 1981 and 1991. We describe the influences of some demographic variables on women’s drinking and identify personal and social risk factors for problem drinking.^4^ Finally, we suggest implications for prevention of alcohol problems and make some recommendations for future research on women’s drinking.

## Trends in Women’s Drinking

Not only have drinking patterns among women varied across different periods in history, but society’s concern about women’s drinking has varied even more (Fillmore 1984). During the 1970’s, for example, media reports and public perception presumed that drinking among North American women was increasing dramatically and approaching the drinking levels of men.

To test the notion that women’s drinking levels were converging on those of men over the last two decades, Hilton (1988) compared 11 surveys conducted between 1964 and 1984. The study did not detect any major variations in alcohol consumption, although there were fluctuations in women’s drinking behavior within specific age groups, and the overall drinking (nonabstention) rate was highest in the most recent survey. Yet there was no evidence for a convergence of drinking rates between men and women. In all age groups and across all surveys, men had significantly higher rates of drinking and heavy drinking than women.

In an analysis of a 1983 and a 1988 national survey, Williams and DeBakey (1992) also detected no convergence of drinking behavior of women and men. But contrary to Hilton’s study (1988), they observed a trend toward reduced alcohol consumption among both women and men. This trend is consistent with declining alcohol sales in the United States during the 1980’s (Williams et al. 1992). Among women, the study found increased abstinence and/or decreased heavy drinking in all age groups and in several other demographic subgroups.

Because Hilton’s and Williams and DeBakey’s studies used somewhat different criteria to assess drinking and cannot be compared directly, we reanalyzed the 13 surveys used in their studies, two 1990 surveys, and our own 1991 national survey. The data (table 1) suggest that changes in women’s drinking since 1970 are best described as two trends: a moderate increase in drinking rates for all age groups during the 1970’s, followed by a slight drop during the 1980’s for all age groups (Wilsnack et al. 1993). We did not, however, detect any drastic changes in women’s drinking behavior. Therefore, none of these three studies supports the perception of a dramatic increase in women’s drinking since the 1970’s.

## Longitudinal Analysis of Women’s Drinking, 1981–1991

In addition to looking at time trends of drinking behavior in the general female population, we analyzed changes in drinking patterns and drinking-related problems of individual women over a period of 10 years (Wilsnack 1993). For this study, a sample of 911 women was first interviewed in 1981. In 1986, 300 of these women were reinterviewed, and in 1991, 696 women from the original sample plus a new sample of 403 women were asked the same questions as in 1981. Drinking behavior was analyzed separately for different age groups.

A comparison of results from the 1981 and 1986 surveys indicates that individual drinking behavior fluctuates significantly over time (Wilsnack et al. 1991). This can be illustrated with variations in problem drinking over time. (Problem drinking was defined as fulfilling at least two of the following criteria: average alcohol consumption of two or more standard drinks per day, one or more drinking problems during the past year, or one or more alcohol dependence symptoms during the past year.^5^) Eleven percent of women drinkers who did not report indicators of problem drinking in 1981 showed some of these indicators 5 years later. And one-third of the women who were problem drinkers in 1981 did not fall into that category in 1986.

The greatest changes in drinking behavior were found among women in the youngest age group (21 to 34 years). Fillmore and colleagues (1991) noted a similar trend in their analysis of surveys from 15 countries. This tendency of younger women to move into and out of problem-drinking patterns may be influenced by changes in drinking contexts, drinking partners, and social roles (e.g., employment, marriage, and parenthood) common in this age group.

A comparison of data from the 1981 and 1991 surveys showed modest declines in most measures of drinking behavior. (Some data from the 1981 and 1991 surveys are summarized in table 2.) There were no significant changes in drinking rates (nonabstention) during the study period. The proportion of women who were heavy drinkers, however, was lower in all age groups in 1991 than in 1981. There also was a decline in heavy episodic drinking (six or more drinks per drinking occasion) among women drinkers in most age groups.

Despite the reduction of heavy drinking episodes, women drinkers under age 40 became more likely to report intoxication. This finding may point to the success of alcohol education campaigns in the last decade: women now may be more alert to the intoxicating effects of alcohol and therefore not only may report intoxication more readily but also may reduce their drinking levels because of perceived effects of alcohol.

## Subgroup Variations

Many studies have shown that drinking behavior is influenced by both an individual’s personality and demographic characteristics, such as age, marital status, employment status, and race and ethnicity. Associations between these factors and women’s drinking are discussed below.

Because of necessary limitations to study designs, epidemiologic studies often can analyze relationships between drinking behavior and only a few demographic factors. One has to keep in mind, though, that a person’s drinking behavior is determined by more than just a few characteristics. Only the combination of all demographic factors, together with historical and cohort effects and personal or environmental risk factors (e.g., genetic background and family environment), can explain individual drinking patterns.

### Age

**Figure 1:**
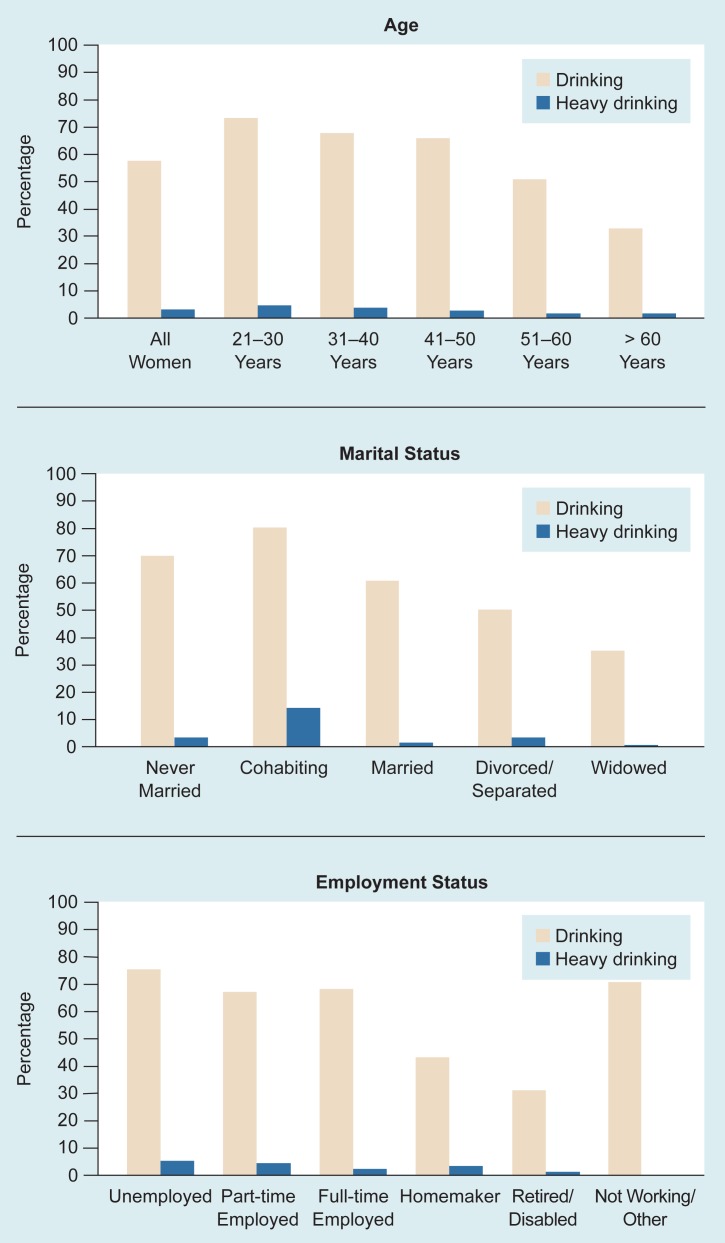
Relationship between the categories of drinking and heavy drinking by women and the demographic characteristics of age, marital status, and employment status. The 1,099 women were interviewed in 1991 as part of the National Longitudinal Study of Women’s Drinking. They were classified as “drinking” if they drank any alcohol in the past 12 months and as “heavy drinking” if they drank two or more standard drinks per day during the past 30 days.

Like gender, age is a strong predictor of drinking behavior. Numerous surveys have shown that younger women have higher rates of heavy drinking and alcohol-related problems than older women (see tables 1 and 2). For instance, in our 1981 and 1991 surveys, women ages 21 to 30 had the highest rates of intoxication, drinking problems, heavy episodic drinking, and alcohol dependence symptoms. Women over 60 had the lowest rates for all these drinking variables (Wilsnack et al. 1984; Wilsnack et al. 1993; see table 2 and figure 1). Similar differences in drinking, heavy drinking, and drinking-related problems across age groups were found in a 1984 national survey (Hilton 1991).

These studies also suggest that younger and older women have different styles of drinking. Younger women tend to engage in heavy episodic drinking that may lead to severe adverse behavioral or social consequences. These can include alcohol-impaired driving and alcohol-related car crashes, both of which have shown dramatic recent increases among younger women (Popkin 1991).

Drinking in older women, however, seems to be characterized by frequent light or moderate drinking. In the 1984 survey, for example, daily drinking was at least as common among women drinkers over 60 as among drinkers in other age groups (Hilton 1991). A 1989 Canadian survey confirmed that among older women drinkers, the frequency of drinking does not decline with age (Health and Welfare Canada 1990). Despite the overall pattern of abstinence or moderation, however, a substantial number of older women develop alcohol-related problems. The combined effects of age and gender create some distinctive treatment and prevention needs among these older women who are problem drinkers (Wilsnack et al. in press).

### Marital Status

Various surveys have shown associations between a woman’s marital status and her drinking behavior (e.g., Clark and Midanik 1982; Wilsnack et al. 1984). These studies found that never-married, divorced, and separated women generally have the highest rates of heavy drinking and drinking-related problems; widowed women, the lowest rates; and married women, intermediate rates.

#### Divorce and Separation

Although in most surveys heavy drinking and drinking-related problems are more prevalent among women who are divorced or separated than among married women (Clark and Midanik 1982; Wilsnack et al. 1984), two studies in the mid-to late 1980’s found somewhat weaker relationships between partnership dissolution and women’s drinking (Health and Welfare Canada 1990; Hilton 1991). In both surveys, the rates of heavier drinking and drinking-related problems among divorced or separated women were similar to those of married women and less than those of women who never had been married. The reasons for this trend remain to be determined.

A few longitudinal studies have tried to determine whether women’s drinking is a cause, consequence, or accompaniment of divorce and separation. Depending on the individual situation, a woman’s drinking probably can be any one of these: an analysis of divorce rates and alcohol consumption in the United States from 1933 to 1984 concluded that divorce rates influence alcohol consumption but not the reverse (Magura and Shapiro 1988). Similarly, survey data from 1982 to 1988 found that women ages 24 to 32 who divorced or separated during the study period reported increased drinking, whereas those who married or remarried showed decreased drinking (Hanna et al. 1993). A British study, in contrast, suggested that heavy drinking contributes to partnership dissolution (Power and Estaugh 1990).

Data from our 1981/1986 longitudinal study indicate that the relationship between divorce and drinking depends on a woman’s history of problem drinking (Wilsnack et al. 1991). For women with no indications of problem drinking in 1981, divorce or separation during the study period were marginally associated with an increase in problem-drinking indicators in 1986. Partnership dissolution, therefore, may be a risk factor in women with no prior alcohol-related problems.

For women who are problem drinkers, however, drinking is a risk factor for subsequent divorce or separation. Women reporting heavy drinking or problem drinking in 1981 were more likely to be divorced or separated in 1986 than were nonproblem drinkers. Somewhat surprisingly, partnership dissolution led to a reduction in problem drinking for these women, perhaps by removing the woman from a distressing relationship in which partnership problems and excessive drinking were mutually reinforcing (Wilsnack and Wilsnack 1993).

#### Cohabitation

Another marital status that is strongly associated with women’s drinking and drinking-related problems is cohabitation. In our 1981 national survey, cohabiting women reported the highest percentage of drinking (100 percent), heavy drinking (18 percent), intoxication, problem consequences, and alcohol dependence symptoms (Wilsnack et al. 1986). A similar result was obtained in the 1991 survey (figure 1), although the drinking and heavy drinking rates both declined over the 10-year study period. Cohabitation also predicted increases in heavy drinking episodes, intoxication, and drinking problems in the 1981/1986 longitudinal study, even after controlling for the women’s ages and previous drinking levels (Wilsnack et al. 1991).

### Employment

During the 1970’s, employment outside the home was considered to affect women’s mental health and drinking behavior adversely, especially when combined with marital or family roles (Johnson 1982). Findings from several surveys conducted in the 1980’s have disputed the notion that employment leads to harmful drinking behavior. Despite the fact that most studies reported higher rates of drinking by women employed outside the home, they found no differences in heavy drinking or adverse drinking consequences between employed women and homemakers (Shore 1992). The 1981/1986 longitudinal study and our 1991 survey also detected no significant relationship between women’s full-time employment and alcohol-related problems (Wilsnack and Wilsnack 1992; figure 1).

Recent survey data are consistent with a new hypothesis that multiple roles, such as family, marriage, and paid employment, can be beneficial for women, reducing their risks of both mental health and drinking problems (Froberg et al. 1986). Potential reasons for this beneficial effect include increased self-esteem and social support, more responsibilities and performance demands, and increased social monitoring, all of which could discourage excessive alcohol use. This hypothesis is supported by data showing that lack of social roles (e.g., being unmarried or without full-time employment) or loss of social roles (e.g., a marriage ending or children leaving home) are associated with higher rates of problem drinking in several age groups of women (Wilsnack and Cheloha 1987).

#### Nontraditional Employment

Whether employment has beneficial or adverse effects on a woman’s drinking behavior depends in part on the kind of job she has. In several recent studies, women working in nontraditional, male-dominated occupations reported increased drinking and/ or adverse drinking consequences. For instance, LaRosa (1990) found that high-ranking women executives were more likely to be drinkers and to drink moderately or heavily than other employed women of comparable age and education. In a study by Wilsnack and Wright (1991), women drinkers in occupations with more than 50 percent male workers scored higher on a problem-drinking index than women in female-dominated occupations.

The influence of gender composition of coworkers on drinking behavior seems to be specific for women. Men’s drinking behavior does not seem to be affected by this factor (Haavio-Mannila 1987; Wilsnack and Wright 1991).

Several interpretations of the relationship between employment that is nontraditional to women and increased drinking by women have been suggested. These include peer influences (i.e., women’s imitation of the male drinking model), more drinking opportunities in nontraditional employment settings, stress related to a minority status in male-dominated occupations, or the use of drinking as a symbolic expression of power and gender equality (Wilsnack and Wilsnack 1993).

### Unwanted Statuses

Factors such as marital status and employment status also can affect a woman’s drinking behavior if they reflect a social position she would prefer *not* to have. Data from our 1981/1986 survey show that an unwanted status (e.g., being unemployed but wanting a job, being married but experiencing marital distress, or being childless but wanting children) can increase the risk of problem drinking in women. An unwanted status is associated with increased alcohol consumption and more adverse drinking consequences. This is especially true for drinking women who had access to alcohol in their home (Wilsnack 1992). In the 1981 survey, the correlation between unwanted statuses and drinking patterns was considerably stronger in women than in a comparison group of men. The reason for this gender difference is not fully understood.

### Race and Ethnicity

Several national surveys have examined ethnic differences in drinking patterns among women and men (e.g., Wilson and Williams 1989), and a national survey conducted in 1984 and 1992 systematically oversampled ethnic minority respondents (Caetano 1991; Herd 1991). In general, these studies have found that among the three major population groups in the United States, white women and men are most likely to drink, African-American women and men are least likely, and Hispanic women and men are in between. In all the groups, men have higher levels of drinking and heavy drinking and higher rates of drinking-related problems and alcohol disorders than women.

Characteristics that influence drinking behavior, such as age, marital status, and employment status, generally have similar effects across ethnic groups, although some exceptions have been noted. For more information on drinking patterns among minority women, see the article by Caetano, pp. 233–241.

One strong predictor of women’s drinking that is valid across ethnic categories is the level of acculturation, that is, adaptation of immigrants to the attitudes, values, and behavior of the population they have entered (Gilbert and Collins in press). Japanese-American women, for example, show less abstention and more light-to-moderate drinking than women in Japan (Kitano et al. 1988). Similarly, heavy drinking increases significantly through successive U.S.-born generations of Mexican-American women (Caetano and Medina Mora 1988).

Most studies of drinking behaviors among ethnic minorities have two drawbacks. First, surveys often include sample sizes of minority respondents that are too small to produce reliable findings. The evaluation of less populous minorities, such as Asian-Americans and American Indians, especially is difficult because of this limitation. Studies oversampling these groups are still rare. Second, the surveys often do not consider differences within ethnic categories.

Studies that do assess heterogeneity within ethnic categories find great variations of drinking behavior. For instance, a study of Asian-Americans in Los Angeles found that Japanese women were less likely to abstain and more likely to drink heavily than were Chinese, Korean, Filipino, or Vietnamese women (Chi et al. 1989). Similar variations were found among different Hispanic subgroups (Caetano 1988) and among tribal groups of American Indians (May 1989).

## Risk Factors for Women’s Drinking

### Influence of Partner’s Drinking

In addition to demographic characteristics, epidemiological studies of women’s drinking have identified other social and personal risk factors for problem drinking. One important predictor is the drinking behavior of partners and husbands. Both clinical studies and general population surveys (e.g., Jacob and Bremer 1986; Kolonel and Lee 1981) have found a positive association between women’s levels of alcohol consumption and those of their partners.

In our 1981 survey of women and a comparison group of men, the perceived frequency of a spouse’s drinking had a stronger association with women’s problem drinking than with men’s problem drinking (Wilsnack and Wilsnack 1993). The notion of a stronger influence of husbands on their wives’ drinking than vice versa is consistent with Haavio-Mannila’s interpretation (1991) that women may imitate the drinking behavior of a “higher-status” male, whether in the family or in the workplace.

Not just conformity but also discrepancies in the drinking patterns of marital partners may increase a person’s risk for problem drinking. In the 1981 survey, wives reported more intoxication and adverse drinking consequences if they perceived discrepancies between their husbands’ drinking and their own than if they perceived drinking patterns as similar (Wilsnack and Wilsnack 1993). A comparable link between discrepant drinking patterns and alcohol problems has been found among men (Gleiberman et al. 1992). It is unclear whether discrepant drinking patterns of spouses or partners reflect conflicts in a relationship, cause them, or both.

### Childhood Sexual Abuse and Relationship Violence

Childhood sexual abuse has been associated with women’s drinking in both clinical studies and national surveys. The prevalence of incest and other childhood sexual abuse is significantly elevated among women in alcoholism treatment (Miller et al. 1993; Russell and Wilsnack 1991). In a 1986 general population survey, we found that twice as many women with a history of problem drinking reported childhood sexual abuse as women without such a history. The experience of childhood sexual abuse also predicted the onset of problem drinking in the 1981–1986 study period (Wilsnack et al. 1991).

Recent research also is exploring potential links between partnership violence toward adult women and their drinking behavior. Several studies have found an association between female drinking and increased victimization in marital violence (Miller and Downs 1993; Kaufman Kantor and Straus 1989). According to a 1992 U.S. survey of alcohol and family violence, a wife’s drinking, whether alone or with her husband, led to more severe violence both by and toward the wife (Kaufman Kantor and Asdigian in press). These studies underscore the need for more attention to relationship violence as both cause and consequence of women’s drinking.

### Sexual Experience

Women experiencing sexual problems or sexual dysfunctions are at increased risk for chronic problem drinking. When we analyzed possible risk factors for women’s drinking in our 1981/1986 longitudinal study, sexual dysfunction was the strongest single predictor of continued problem drinking. The relationship between alcohol and female sexuality is discussed in more detail in the article by Norris, pp. 197–201.

Both women and men expect alcohol to enhance sexual experience, a perception that may increase alcohol consumption. In our 1981 survey, for example, 60 percent of women drinkers (especially heavy drinkers) reported that they felt less sexually inhibited after drinking (Klassen and Wilsnack 1986). These effects were reported most frequently by the heaviest drinkers in the study.

Some surveys (see Cooper 1992) have suggested an association between alcohol consumption and increased risky sexual behavior (i.e., unprotected, with multiple partners, or with partners at high risk for HIV/AIDS). However, most studies to date could not establish a direct causal relationship between drinking and high-risk sexual behavior (Cooper 1992; Leigh 1993). And most women in the 1981 survey reported that drinking did not actually change their sexual behavior (e.g., make them less discriminating in their choice of partners) (Klassen and Wilsnack 1986). Clearly, additional factors, including characteristics of the woman, her partner, and the social situation, influence the connections between women’s drinking and their sexual behavior.

## Conclusions and Future Directions

Analysis of women’s drinking over the past 20 years has suggested a moderate increase in alcohol consumption during the 1970’s, followed by a slight decline in the 1980’s (Hilton 1988; Williams and DeBakey 1992; Wilsnack et al. 1993). Drinking trends among women need to be monitored further to determine whether the decline continues through the 1990’s.

The decline in women’s drinking noted so far seems to be somewhat more gradual or less consistent than the concurrent decline in alcohol sales (Williams et al. 1992; Wilsnack 1993). Further research is needed to determine whether the decrease in women’s alcohol consumption is happening more slowly than the decrease in men’s and, if so, why.

Similar attention must be paid to the changes in drinking patterns among minority women. So far, the trend toward reduced alcohol consumption seems to be clearest for white women and less pronounced among African-Americans and Hispanics (Midanik and Clark 1992; Caetano and Kaskutas 1993). Future studies should address this issue by over-sampling minority women, especially women who are heavy drinkers. This is particularly important for minorities, such as Asian-Americans or American Indians, who have been underrepresented in surveys until now.

The overall decline in women’s drinking should not lead to alcohol research shifting away from women’s issues for at least two reasons. First, there are some indications that drinking problems and alcohol disorders may still be increasing in some age groups. Future research must determine whether the higher rates of alcohol problems among younger women found in some studies (e.g., Robins et al. 1988) are a result of increased recognition of alcohol problems and a greater willingness to report them or whether the prevalence of these disorders is actually increasing.

Second, if generally decreasing alcohol consumption leads to a shift in drinking norms and reduced tolerance toward people with alcohol-related problems (Room 1991), it could become even more difficult than it is at present for women who are problem drinkers to acknowledge and overcome their problems. Such a development would increase the need for gender-sensitive prevention and treatment services for these women.

Drinking behavior in women clearly is related to demographic characteristics, such as age, marital status, and employment status. Therefore, future prevention and treatment research should focus on specific subgroups as well as the female population in general. Recent research already has identified and addressed some subgroup-specific characteristics of drinking behavior. More detailed studies are needed to answer questions such as the following and to translate the findings into new prevention strategies:

- How can heavy episodic drinking and alcohol-impaired driving be reduced in young women?
- How do divorce and separation affect women’s drinking behavior? When do they have beneficial effects and when do they have adverse effects?
- How can the apparent adverse effects of cohabitation on drinking behavior be prevented or addressed?
- When are multiple social roles harmful or protective for women’s drinking behavior?
- What effect does the lack of social roles have on drinking? How can this lack be counteracted?

The answers to these questions can lead to prevention and treatment approaches that target specifically the shared or distinct characteristics of both women’s and men’s drinking behavior and thus improve the effectiveness of intervention for both genders.

## Figures and Tables

**Table 1:** Timeline of Drinking and Heavy Drinking Among Women, 1981–1991, by Age Group (in percent)

Age Group (years)	HP 1971	HP 1972	HP 1973	HP 1973	HP 1974	ORC 1975	RAC 1976	SRG 1979	UND 1981	NHIS 1983	ARG 1984	NHIS 1988	ARG 1990	NHIS 1990	UND 1991
21–34															
Drinkers	71	67	72	65	71	68	71	77	71	79	73	71	73	76	74
Heavy drinkers	6	4	5	3	6	5	4	5	6	4	7	3	3	2	4
35–49															
Drinkers	64	56	63	55	65	57	73	65	73	74	64	68	64	74	65
Heavy drinkers	5	4	8	5	6	3	3	8	8	5	6	4	2	2	3
50–64															
Drinkers	47	44	43	50	49	48	50	49	53	67	62	59	57	66	52
Heavy drinkers	5	4	5	4	4	1	3	3	5	4	4	4	2	2	2
65 and Over															
Drinkers	26	42	29	28	36	32	37	40	33	53	44	45	32	52	29
Heavy drinkers	0	5	2	2	2	1	0	2	2	3	2	2	1	2	1

NOTE: HP = Harris Poll; ORC = Opinion Research Corporation; RAC = Response Analysis Corporation; SRG = Social Research Group (later Alcohol Research Group); -UND = University of North Dakota; NHIS = National Health Interview Survey; ARG = Alcohol Research Group. -

SOURCE: Johnson et al. 1977; Wilsnack et al. 1984; Williams and DeBakey 1992 (reanalyzed by S.F. DeBakey); Hilton 1988; Midanik and Room 1992 (reanalyzed by -L.T. Midanik); Williams et al. 1993 (reanalyzed by S.F. DeBakey).

**Table 2:** National Longitudinal Study of Women’s Drinking: Comparison of 1981 and 1991 Drinking Patterns Among Women of Different Age Groups (in percent, weighted)

Age Group (years)	Year	Abstainers	Heavy Drinkers	Frequency > 1/month	Drinks/ Drinking Day > 1	Days of ≥ 6 Drinks ≥ 1	Days Felt Drunk > 1	Problem Consequences ≥ 1	Dependence Symptoms ≥ 1	*n*
21–30 -	1981	24.3	6.8	39.9	56.1	40.7	38.1	32.8	23.6	275
	1991	27.1	4.4	36.7*^1^	49.4^†^	27.5^§^	40.9	26.5^†^	22.1	405
31–40 -	1981	29.8	4.2	35.0	41.5	24.0	21.2	13.8	9.5	194
	1991	31.1	3.5	26.3*	42.5	17.1^†^	34.6^†^	17.3	10.5	235
41–50 -	1981	32.8	9.9	37.9	37.1	22.5	16.7	13.2	10.8	147
	1991	33.6	2.3*	22.6^†^	25.6^†^	9.5^‡^	13.1	9.9	8.7	162
51–60 -	1981	44.5	5.2	29.2	29.4	14.3	9.5	7.6	4.0	132
	1991	49.1	1.4	22.6*	24.6*	10.4*	12.3	6.8	3.7	122
61–70 -	1981	64.0	4.1	23.5	18.4	11.8	6.1	3.3	1.7	104
	1991	60.1	1.1	11.2	14.4	5.8^†^	3.6	3.1	1.1	99
71+ -	1981	67.0	2.4	15.6	8.1	0.9	0.9	2.4	0.5	59
	1991	76.3	2.6	12.3	7.3	2.6	1.4	2.1	2.5	76
Total	1981	38.9	5.7	32.5	36.6	22.8	19.2	15.1	10.6	911
Sample	1991	42.2	2.7	23.7	30.5	13.8	21.0	12.8	9.5	1,099

1 Statistical significance = *p < 0.10; ^†^*p* < 0.05; ^‡^*p* < 0.01; ^§^*p* < 0.001. -

NOTE: In 1981, 911 women were interviewed about their drinking patterns in the previous 12 months. In 1991, 696 of these women plus a new group of 403 women were asked -the same questions as in 1981. -

## Footnotes

1 A standard drink is defined as 12 fluid ounces of beer, 5 fluid ounces of wine, or 1.5 fluid ounces of distilled spirits and contains about 0.5 fluid ounce of pure alcohol.

2 In longitudinal surveys, the same people are interviewed two or more times after certain time intervals.

3 The terms “drinking-related problems,” “alcohol-related problems,” “alcohol problems,” and “drinking problems” refer to drinking patterns that have caused negative social, personal, or medical consequences.

4 Terms such as “problem drinking,” “frequent light drinking,” or “moderate drinking” are defined as they are used

5 These criteria do not attempt to approximate a clinical diagnosis of alcohol abuse or alcohol dependence; they merely identify a group of women who may be experiencing problems related to their use of alcohol. The threshold for each criterion is relatively low. However, requiring at least two of the three criteria identifies about 10 percent of the women sampled as problem drinkers, a percentage that falls in the middle of estimated rates of problem drinking among U.S. women during the two decades preceding the 1981 survey (Ferrence 1980).

## References

[b1-arhw-18-3-173] American Psychiatric Association (1994). Diagnostic and Statistical Manual of Mental Disorders.

[b2-arhw-18-3-173] Caetano R (1988). Alcohol use among Hispanic groups in the United States. American Journal of Drug and Alcohol Abuse.

[b3-arhw-18-3-173] Caetano R, Clark WB, Hilton ME (1991). Findings from the 1984 National Survey of Alcohol Use among U.S. Hispanics. Alcohol in America: Drinking Practices and Problems.

[b4-arhw-18-3-173] Caetano R, Kaskutas LA Longitudinal Changes in Drinking Patterns Among Whites, Blacks, and Hispanics: 1984–1992.

[b5-arhw-18-3-173] Caetano R, Medina Mora ME (1988). Acculturation and drinking among people of Mexican descent in Mexico and the United States. Journal of Studies on Alcohol.

[b6-arhw-18-3-173] Chi L, Lubben JE, Kitano HHL (1989). Differences in drinking behavior among three Asian-American groups. Journal of Studies on Alcohol.

[b7-arhw-18-3-173] Clark WB, Midanik L (1982). Alcohol use and alcohol problems among U.S. adults: Results of the 1979 national survey. Alcohol Consumption and Related Problems.

[b8-arhw-18-3-173] Cooper ML (1992). Alcohol and increased behavioral risk for AIDS. Alcohol Health & Research World.

[b9-arhw-18-3-173] Ferrence RG, Kalant OJ (1980). Sex differences in the prevalence of problem drinking. Alcohol and Drug Problems in Women. Research Advances in Alcohol and Drug Problems.

[b10-arhw-18-3-173] Fillmore KM, Wilsnack SC, Beckman LJ (1984). When angels fall: Women’s drinking as cultural preoccupation and as reality. Alcohol Problems in Women: Antecedents, Consequences, and Intervention.

[b11-arhw-18-3-173] Fillmore KM, Hartka E, Johnstone BM, Leino EV, Motoyoshi M, Temple MT (1991). A meta-analysis of life course variation in drinking: The Collaborative Alcohol-Related Longitudinal Project. British Journal of Addiction.

[b12-arhw-18-3-173] Froberg D, Gjerdingen D, Preston M (1986). Multiple roles and women’s health: What have we learned?. Women and Health.

[b13-arhw-18-3-173] Gilbert MJ, Collins RL, Wilsnack RW, Wilsnack SC Ethnic variation in women’s and men’s drinking. Gender and Alcohol.

[b14-arhw-18-3-173] Gleiberman L, Harburg E, DiFrancisco W, Schork A (1992). Familial transmission of alcohol use. Part V. Drinking patterns among spouses. Tecumseh, Michigan. Behavior Genetics.

[b15-arhw-18-3-173] Gomberg ESL, Nirenberg TD (1993). Women and Substance Abuse.

[b16-arhw-18-3-173] Haavio-Mannila E (1987). Alkohol, Arbete och Familij—En Jamforelse Mellan Man och Kvinnor.

[b17-arhw-18-3-173] Haavio-Mannila E Impact of Colleagues and Family Members on Female Alcohol Use.

[b18-arhw-18-3-173] Hanna EZ, Faden VB, Harford CT (1993). Marriage: Does it protect young women from alcoholism?. Journal of Substance Abuse.

[b19-arhw-18-3-173] Health and Welfare Canada (1990). National Alcohol and Other Drugs Survey (1989): Highlights Report.

[b20-arhw-18-3-173] Herd D, Clark WB, Hilton ME (1991). Drinking patterns in the black population. Alcohol in America: Drinking Practices and Problems.

[b21-arhw-18-3-173] Hill SY, Galanter M Mental and physical health consequences of alcohol use in women. Recent Developments in Alcoholism. Vol. 12. Alcoholism and Women: The Effect of Gender.

[b22-arhw-18-3-173] Hilton ME (1988). Trends in U.S. drinking patterns: Further evidence from the past 20 years. British Journal of Addiction.

[b23-arhw-18-3-173] Hilton ME, Clark WB, Hilton ME (1991). The demographic distribution of drinking patterns in 1984. Alcohol in America: Drinking Practices and Problems.

[b24-arhw-18-3-173] Jacob T, Bremer DA (1986). Assortative mating among men and women alcoholics. Journal of Studies on Alcohol.

[b25-arhw-18-3-173] Johnson PB (1982). Sex differences, women’s roles and alcohol use: Preliminary national data. Journal of Social Issues.

[b26-arhw-18-3-173] Kaufman Kantor G, Asdigian NL, Wilsnack RW, Wilsnack SC Gender differences in alcohol related spousal aggression. Gender and Alcohol.

[b27-arhw-18-3-173] Kaufman Kantor G, Straus MA (1989). Substance abuse as a precipitant of wife abuse victimizations. American Journal of Drug and Alcohol Abuse.

[b28-arhw-18-3-173] Kessler RC, McGonagel KA, Zhao S, Nelson CH, Hughes M, Eshleman S, Wittchen HU, Kendler KS (1994). Lifetime and 12-month prevalence of DSM–III–R psychiatric disorders in the United States. Archives of General Psychiatry.

[b29-arhw-18-3-173] Kitano HHL, Lubben JE, Chi L (1988). Predicting Japanese American drinking behavior. International Journal of the Addictions.

[b30-arhw-18-3-173] Klassen AD, Wilsnack SC (1986). Sexual experience and drinking among women in a U.S. national survey. Archives of Sexual Behavior.

[b31-arhw-18-3-173] Kolonel LN, Lee J (1981). Husband-wife correspondence in smoking, drinking, and dietary habits. American Journal of Clinical Nutrition.

[b32-arhw-18-3-173] LaRosa JH (1990). Executive women and health: Perceptions and practices. American Journal of Public Health.

[b33-arhw-18-3-173] Leigh BC (1993). Alcohol consumption and sexual activity as reported with a diary technique. Journal of Abnormal Psychology.

[b34-arhw-18-3-173] Magura M, Shapiro E (1988). Alcohol consumption and divorce: Which causes which?. Journal of Divorce.

[b35-arhw-18-3-173] May PA, Watts TD, Wright R (1989). Alcohol abuse and alcoholism among American Indians: An overview. Alcoholism in Minority Populations.

[b36-arhw-18-3-173] Midanik LT, Clark WB The Demographic Distribution of U.S. Drinking Patterns in 1990: Description and Trends From 1984.

[b37-arhw-18-3-173] Miller BA, Downs WR (1993). The impact of family violence on the use of alcohol by women. Alcohol Health & Research World.

[b38-arhw-18-3-173] Miller BA, Downs WR, Testa M (1993). Interrelationships between victimization experiences and women’s alcohol use. Journal of Studies on Alcohol.

[b39-arhw-18-3-173] Popkin CL (1991). Drinking and driving by young females. Accident Analysis and Prevention.

[b40-arhw-18-3-173] Power C, Estaugh V (1990). The role of family formation and dissolution in shaping drinking behaviour in early adulthood. British Journal of Addiction.

[b41-arhw-18-3-173] Robins LN, Helzer JE, Przybeck TR, Regier DA, Rose RM, Barrett JE (1988). Alcohol disorders in the community: A report from the ECA. Alcoholism: Origins and Outcome.

[b42-arhw-18-3-173] Room R, Clark WB, Hilton ME (1991). Cultural changes in drinking and trends in alcohol problem indicators: Recent U.S. experience. Alcohol in America: Drinking Practices and Problems.

[b43-arhw-18-3-173] Russell SA, Wilsnack SC, Roth P (1991). Adult survivors of childhood sexual abuse: Substance abuse and other consequences. Alcohol and Drugs Are Women’s Issues. Vol. 1: A Review of the Issues.

[b44-arhw-18-3-173] Shore ER (1992). Drinking patterns and problems among women in paid employment. Alcohol Health & Research World.

[b45-arhw-18-3-173] Williams GD, DeBakey SF (1992). Changes in levels of alcohol consumption: United States, 1983–1988. British Journal of Addiction.

[b46-arhw-18-3-173] Williams GD, Stinson FS, Clem D, Noble J (1992). Surveillance Report #23: Apparent Per Capita Alcohol Consumption: National, State, and Regional Trends, 1977–1990.

[b47-arhw-18-3-173] Wilsnack RW (1992). Unwanted statuses and women’s drinking. Journal of Employee Assistance Research.

[b48-arhw-18-3-173] Wilsnack RW, Cheloha R (1987). Women’s roles and problem drinking across the lifespan. Social Problems.

[b49-arhw-18-3-173] Wilsnack RW, Wilsnack SC (1992). Women, work, and alcohol: Failures of simple theories. Alcoholism: Clinical and Experimental Research.

[b50-arhw-18-3-173] Wilsnack RW, Wright SI Women in Predominantly Male Occupations: Relationships to Problem Drinking.

[b51-arhw-18-3-173] Wilsnack RW, Wilsnack SC, Klassen AD (1984). Women’s drinking and drinking problems: Patterns from a 1981 national survey. American Journal of Public Health.

[b52-arhw-18-3-173] Wilsnack RW, Harris TR, Wilsnack SC Changes in U.S. Women’s Drinking: 1981–1991.

[b53-arhw-18-3-173] Wilsnack SC Patterns and Trends in Women’s Drinking: Recent Findings and Some Implications for Prevention.

[b54-arhw-18-3-173] Wilsnack SC, Beckman LJ (1984). Alcohol Problems in Women: Antecedents, Consequences, and Intervention.

[b55-arhw-18-3-173] Wilsnack SC, Wilsnack RW, Gomberg ESL, Nirenberg TD (1993). Epidemiological research on women’s drinking: Recent progress and directions for the 1990s. Women and Substance Abuse.

[b56-arhw-18-3-173] Wilsnack SC, Wilsnack RW, Klassen AD (1986). Epidemiological research on women’s drinking, 1978–1984. Women and Alcohol: Health-Related Issues.

[b57-arhw-18-3-173] Wilsnack SC, Klassen AD, Schur BE, Wilsnack RW (1991). Predicting onset and chronicity of women’s problem drinking: A five-year longitudinal analysis. American Journal of Public Health.

[b58-arhw-18-3-173] Wilsnack SC, Vogeltanz ND, Diers LE, Wilsnack RW, Beresford TP, Gomberg ESL Drinking and problem drinking in older women. Alcohol and Aging.

[b59-arhw-18-3-173] Wilson RW, Williams GD (1989). Alcohol use and abuse among U.S. minority groups: Results from the 1983 National Health Interview Survey. Alcohol Use Among U.S. Ethnic Minorities.

